# Acute Unprovoked Pulmonary Embolism As the First Presentation in Undiagnosed Acromegaly

**DOI:** 10.7759/cureus.94452

**Published:** 2025-10-13

**Authors:** Saad Muhammad, Aly Barakat, Annzel Jais, Sumaya Akter, Yorissa Padayachee

**Affiliations:** 1 Department of Cardiology, Medway NHS Foundation Trust, Gillingham, GBR

**Keywords:** acromegaly comorbidities, acromegaly complications, acromegaly symptoms, acute pulmonary embolism, coagulation disorders, risk factors cardiovascular diseases, venous thromboembolism (vte)

## Abstract

The association between acromegaly and hypercoagulability may cause undiagnosed patients to present with venous thromboembolism. We present a man in his late 50s who attended the emergency department on account of pleuritic chest pain and shortness of breath. His past medical history included epilepsy (with a previous temporal lobe lobectomy) and prediabetes. Computed tomography pulmonary angiogram established a diagnosis of acute bilateral pulmonary emboli involving the right and left subsegmental pulmonary arteries, which warranted further investigation for a cause. No obvious provoking factors were found, and further CT imaging did not reveal any underlying malignancy. As the patient was noted on his admission clerking to have coarse facial features with enlarged hands and feet, endocrine evaluation was undertaken; elevated insulin-like growth factor-1, along with a positive oral glucose tolerance test, confirmed the diagnosis of acromegaly. The patient was discharged with anticoagulation and referred to the thrombosis clinic alongside an outpatient MRI of his pituitary, the findings of which were consistent with a pituitary macroadenoma.

## Introduction

Acromegaly is a rare condition characterized by excessive secretion of growth hormone (GH) and insulin-like growth factor 1 (IGF-1) [1]. The prevalence of acromegaly ranges between 2.8 and 13.7 per 100,000 individuals, with an annual incidence between 0.2 and 1.1 cases per 100,000 [2]. The time between the onset of initial symptoms and diagnosis is often delayed, ranging up to 10 years [3].

Most cases of acromegaly are due to somatotropinomas, with the majority occurring sporadically. Some syndromes and familial diseases may result in acromegaly, but these are less common [4].

Common clinical manifestations of acromegaly are multisystem due to excess circulating GH and IGF-1, which can cause headaches, acral enlargement, skin and soft tissue changes (prognathism, macroglossia, prominent forehead creasing, jaw malocclusion, hypertrophy of frontal bones, and an enlarged nose), carpal tunnel syndrome, arthralgia, snoring, and visual field defects [1].

Venous thromboembolism (VTE) progressing to pulmonary embolism (PE) is a life-threatening condition that requires immediate treatment. Typical presentations include dyspnea, chest pain, sinus tachycardia, and decreased oxygen levels. VTE is a common cause of hospital admission with a yearly incidence of one to two in every 1,000 adults [5].

Previous studies have described the association with hypercoagulability and acromegaly; in particular, these individuals have been shown to have higher fibrinogen levels, lower protein C and S activity, and enhanced platelet function [6-8]. Although there have been previous reports of VTE in patients with acromegaly [9-11], this relationship remains less frequently mentioned in medical literature.

## Case presentation

Initial presentation

A man in his late 50s presented to the Emergency Department with a two-week history of pleuritic left-sided chest pain radiating to the left shoulder, which started following lifting a gazebo. The pain was associated with worsening shortness of breath and an acutely reduced exercise tolerance of a single flight of stairs. He had a history of epilepsy managed with phenytoin after he underwent a previous temporal lobectomy. Aside from this and a diagnosis of prediabetes, there was no other significant medical or family history. He denied smoking cigarettes, using recreational drugs, or consuming any alcohol.

On admission, he was found to be tachypneic with a respiratory rate of 23 breaths/minute, an oxygen saturation level of 96% on room air, and a heart rate of 102 beats/minute. It was noted that he had coarse facial hair, large hands, and pins-and-needle sensations occurring at night in both hands. Tinel’s and Phalen’s tests were both found to be positive, indicating bilateral carpal tunnel syndrome. The rest of his neurological examination was normal, and importantly, he did not have any visual field defects.

Initial investigations

**Figure 1:**
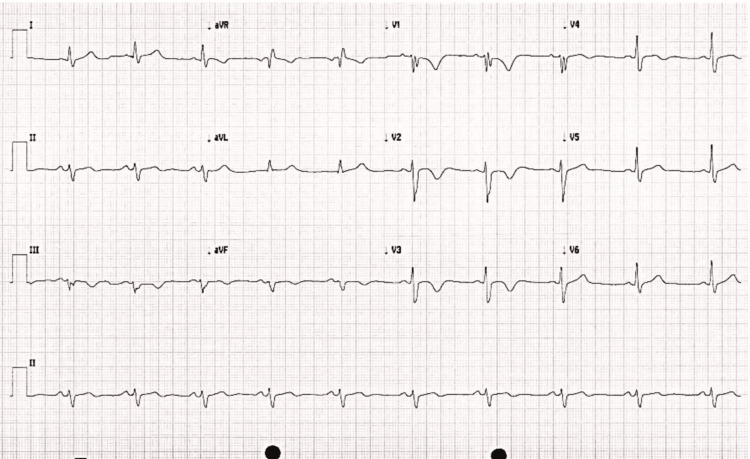
12-lead admission electrocardiogram Standard 12-lead electrocardiogram demonstrating sinus rhythm at 58 beats/minute, with T-wave inversion in the anteroseptal leads and a right bundle branch block aVR: augmented vector right; aVL: augmented vector left; aVF: augmented vector foot

Initial blood investigation results were unremarkable. An electrocardiogram (ECG; Figure 1) revealed a right bundle branch block with T-wave inversion in the anterior and septal leads. This was accompanied by an initial high sensitivity troponin level of 101 ng/L. The initial diagnosis was a probable non-ST-elevation myocardial infarction event given the new ECG findings (particularly the T wave inversion in V3 and V4) and the raised troponin levels. Repeated troponin results came at a level of 94 ng/L. He underwent a coronary angiogram in view of the new ECG changes and mild troponin rise of 101 ng/L, which showed unobstructed coronary arteries. Subsequently, given the normal coronary angiogram, his acute coronary syndrome treatment was stopped.

Differential diagnosis

**Figure 2:**
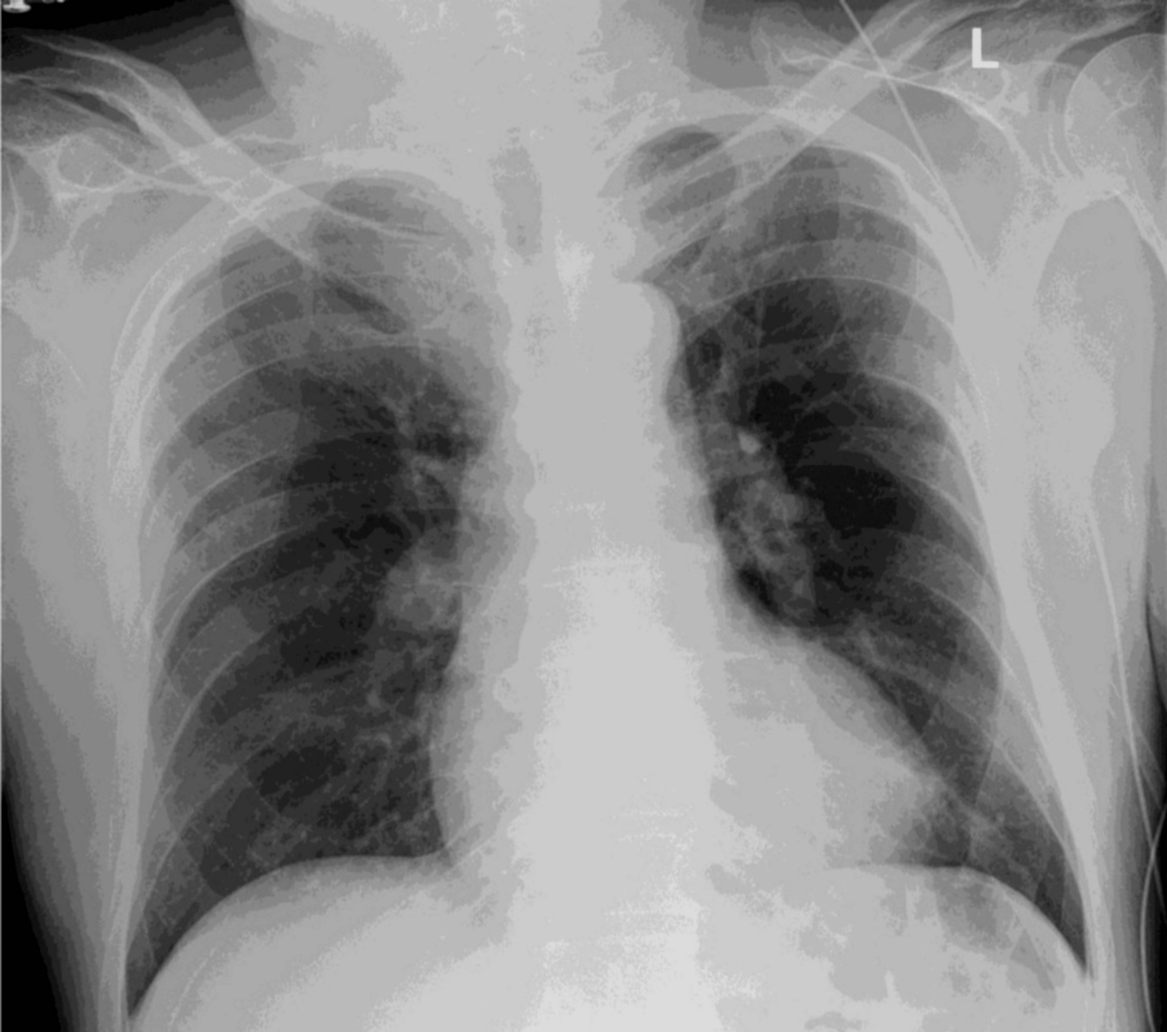
Initial chest radiograph Anteroposterior chest X-ray showing no signs of pulmonary congestion or pleural effusion. Lung fields appear clear, and bony thoracic structures are unremarkable. The cardiomediastinal and hilar shadows appear normal

**Figure 3:**
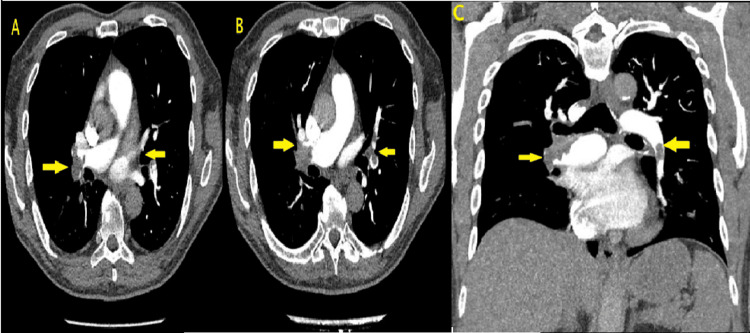
CTPA showing bilateral pulmonary emboli Axial (A,B) and coronal (C) views of the pulmonary arteries showing bilateral pulmonary emboli in the right and left subsegmental pulmonary arteries CTPA: computed tomography pulmonary angiogram

**Figure 4:**
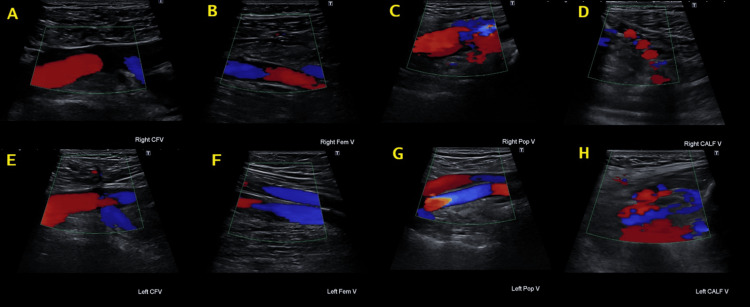
Ultrasound Doppler scan of lower limbs showing no evidence of thrombosis Ultrasound Doppler scans showing the right common femoral vein (A), right femoral vein (B), right popliteal vein (C), right calf vein (D), left common femoral vein (E), left femoral vein (F), left popliteal vein (G), and left calf vein (H). There is no evidence of thrombosis seen in any of the images CFV: common femoral vein

**Figure 5:**
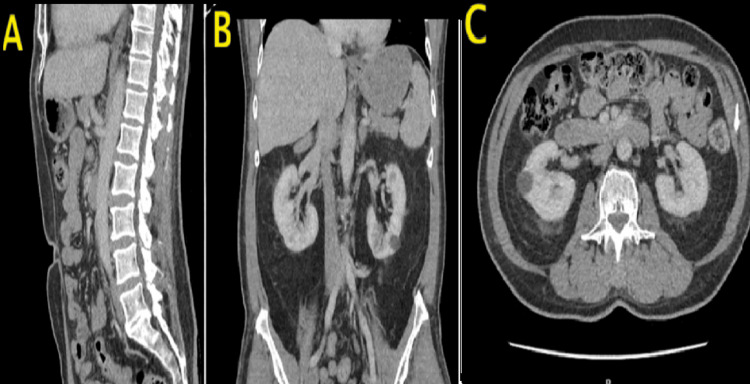
CTAP showing no evidence of malignancy Sagittal (A), coronal (B), and axial (C) views of the abdomen and pelvis showing no evidence of malignancy CTAP: CT scan of the abdomen and pelvis

Twenty-four hours after admission, the patient developed a new oxygen requirement with a normal chest X-ray (Figure 2). This warranted an urgent computed tomography pulmonary angiogram, which confirmed the diagnosis of bilateral acute PE involving the right and left subsegmental arteries without any features of right heart strain or malignancy, as shown in Figures 3A-3C. Lower limb ultrasound Doppler showed no radiological signs of deep vein thrombosis bilaterally (Figure 4). A further CT scan of the abdomen and pelvis ruled out any evidence of underlying malignancy (Figure 5). To note, the patient denied any periods of immobilization or trauma.

The patient was treated for an unprovoked PE and anticoagulated. His anticoagulation had to be discussed with neurology, given that he was on phenytoin (a CYP450 inducer), possibly resulting in reduced efficacy of a direct oral anticoagulant. Following a thorough discussion with the hematology and the neurology teams, alongside counseling the patient, a decision was made to initiate warfarin.

Endocrine investigations and outcome

**Figure 6:**
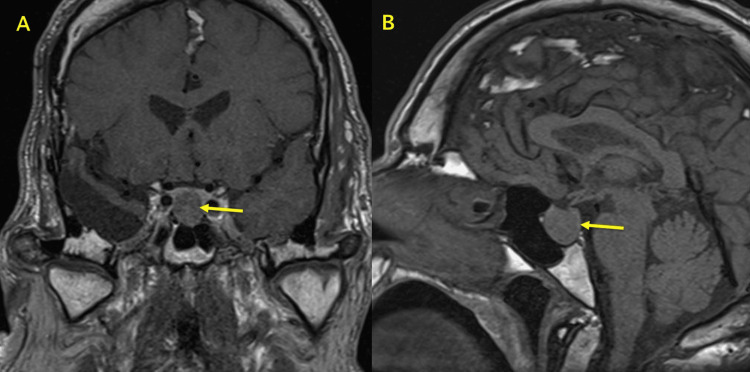
MRI showing pituitary adenoma Coronal (A) and sagittal (B) MRI images demonstrating a pituitary macroadenoma measuring approximately 17 mm craniocaudally and 16 mm anteroposteriorly. The lesion does not encase the carotid arteries, with preserved pituitary stalk position and a normal-appearing optic chiasm. A previous right temporal lobe lobectomy can also be seen in the sagittal (B) image

Given the coarse features noted previously on physical examination and with the diagnosis of an acute unprovoked PE in mind, a further workup for acromegaly was performed. This showed an elevated IGF-1 level of 89.6 nmol/L and a low testosterone level of 6.7nmol/L (Table 1), and a positive oral glucose tolerance test (Table 2). A pituitary MRI scan demonstrated a pituitary macroadenoma measuring 17 mm craniocaudally and 16 mm anterior-posteriorly, as shown in Figures 6A, 6B. The patient was booked for an outpatient endocrinology follow-up for the new diagnosis of acromegaly.

**Table 1:** Laboratory results GFR: glomerular filtration rate; INR: international normalized ratio; ALT: alanine aminotransferase; IGF-1: insulin-like growth factor 1; TSH: thyroid stimulating hormone; LH: luteinizing hormone

Test	Results	Unit	Reference range
Estimated GFR	86	mL/minute/1.73 m^2^	-
Creatinine	80	umol/L	59-104
Sodium	143	mmol/L	133-146
Potassium	3.4	mmol/L	3.5-5.3
Albumin	43	g/L	35-50
Total bilirubin	12	umol/L	0-21
Alkaline phosphatase	87	U/L	30-130
ALT	14	U/L	<50
Total leucocyte count	5.6	10^9^/L^*^	4.0-11.0
Hemoglobin	156	g/L	130-170
Hematocrit	0.47	L/L	0.40-0.50
Platelet count	244	10^9^/L^*^	150-410
Fibrinogen	5.4	g/L	2.8-4.7
INR	1.6	ratio	0.8-1.2
Prothrombin time	17.4	seconds	9.4-12.5
IGF-1	>157.2	nmol/L	6.2-25.2
TSH	1.80	mIU/L	0.30-4.80
LH	2.4	IU/L	1.5-9.3
Prolactin	229	mIU/L	56-278
Random cortisol	305	nmol/L	-

**Table 2:** Oral glucose tolerance test results GH: growth hormone; FPG: fasting plasma glucose

Time (minutes)	Plasma glucose (mmol/L)	GH (µg/L)
	Reference range: <5.6 (normal), 5.6-6.9 (impaired FPG), ≥7.0 (diabetes)	Reference range: <1 µg/L (suppressed)
Fasting (0)	4.5	14.8
30	7.5	17.6
60	9.7	18.7
90	8	13.5
120	7	14

## Discussion

Previous studies have reported an association between acromegaly and an increased risk of VTE [12-14]. A Danish retrospective nationwide cohort study by Dal et al. found patients with acromegaly have a higher risk of VTE in comparison with the general population, with a hazard ratio of 2.3 for VTE events [12]. This elevated risk of complications was found to be higher compared to the general population before the diagnosis of acromegaly was made, suggesting that chronic exposure to excess GH and IGF-1 contributes to a prothrombotic state.

From a mechanistic perspective, patients with acromegaly exhibit hypercoagulability characterized by increased levels of fibrinogen, factor VIII, and enhanced thrombin generation, resulting in denser and more stable fibrin clots, which may predispose these patients to thrombosis [13,15]. Erem et al. also showed an inverse correlation between GH and tissue factor pathway inhibitor, as well as increased plasminogen activator inhibitor-1 levels [16]. Multiple studies have also reported evidence of decreased protein C and S activity, further contributing to a prothrombotic picture [7,8,16].

A study by Landin-Wilhelmsen et al. even showed a reduction in elevated fibrinogen levels following the treatment of patients with acromegaly [6]. These findings highlight the importance of prompt recognition and treatment of acromegaly to reduce patient risk of cardiovascular disease.

Endothelial dysfunction and systemic inflammation are both more pronounced in active or uncontrolled acromegaly [17,18]. These biochemical and functional changes are consistent with the observed increase in cardiovascular morbidity and mortality in this population, particularly from cardiometabolic complications such as diabetes mellitus, heart failure, and VTE [12,14,19,20].

These findings support the hypothesis of a hypofibrinolytic and prothrombotic disposition in patients with acromegaly. Recent multicenter cohort data by Isand et al. furthers this relationship by showing the cumulative incidence of VTE in acromegaly patients is higher than in patients with nonfunctioning pituitary adenomas, with a history of diabetes mellitus further increasing this risk [14].

Clinicians should, however, be careful not to anchor their diagnosis and be willing to explore other potential causes for the development of VTE in patients with acromegaly. As with our case presentation, provoking factors such as malignancy and immobility should be ruled out, and ideally, further exploration of laboratory findings related to coagulation should be conducted, particularly given the biochemical evidence from previous studies [7,8,13,15,16].

In summary, there is robust clinical and biochemical evidence that supports the association between acromegaly and prothrombotic states, with an increased risk of VTE, PE, and deep vein thrombosis, mediated by multiple hemostatic and endothelial abnormalities [6-8,12-16,19]. Although these data exist, the diagnosis of acromegaly is not usually considered a risk factor for VTE across practice.

## Conclusions

In this case report, we reemphasize that a diagnosis of acromegaly should prompt clinicians to consider VTE as a differential if symptoms are suggestive, as these patients may be at an increased risk of thrombosis. In addition, a diagnosis of acromegaly should also not deter clinicians from investigating other provoking factors.
